# Empagliflozin Attenuates Cardiac Dysfunction in Rat Model of Metabolic Syndrome: Evaluating Role of the Cardiac Renin–Angiotensin System

**DOI:** 10.3390/biomedicines14071533

**Published:** 2026-07-08

**Authors:** Reihaneh Ghasemi Tarie, Alireza Esteghamati, Kamran Rakhshan, Sadaf Esteghamati, Mansoor Keshavarz

**Affiliations:** 1 Department of Physiology, School of Medicine, Tehran University of Medical Sciences, Tehran 1461884513, Iran; 2 Endocrinology and Metabolism Research Center (EMRC) Vali-Asr Hospital, Tehran University of Medical Sciences, Tehran 1411713135, Iran; 3 School of Business, University of La Verne, La Verne, CA 91750, USA

**Keywords:** metabolic syndrome, cardiometabolic syndrome, sodium-glucose transporter 2 inhibitors, renin–angiotensin system, empagliflozin, cardiac renin–angiotensin system

## Abstract

**Background:** Cardiometabolic syndrome is a cardiovascular disease characterized by metabolic dysregulation, with obesity triggering overactivation of the cardiac Renin–Angiotensin System (RAS). This leads to pathological cardiac changes and dysfunction. Empagliflozin (EMPA) modulates local RAS components in the kidney and liver, but its role in regulating cardiac RAS needs further study. **Methods:** Twenty-four male Wistar rats were separated into the following two groups: (1) control and (2) metabolic syndrome (MS) fed a high-fat diet, and after 8 weeks, half of each group was treated with EMPA (10 mg/kg) for 8 subsequent weeks. Finally, the animals underwent echocardiography, and under sodium thiopental anesthesia, blood samples were taken for FBS and lipid profile measurement. Finally, the left ventricle was isolated and used to measure the levels of proteins in the RAS pathway, including AngII (Angiotensin2), AT1R (Angiotensin2type1receptor), AT2R (Angiotensin2type2 receptor), and downstream pathway proteins pERK1/2 (Phosphorylated Extracellular Signal-Regulated Kinase1/2), NHE1 (Na+/H+ Exchanger1), NCX (Na+/Ca^2^+Exchanger), and NLRP3 (NOD-like-receptor-protein3) by Western blot, as well as ROS (reactive oxygen species) levels by ELISA. **Results**: EMPA treatment in MS significantly decreased FBS, TG, and LDL, increased HDL, and improved cardiac function. It was also associated with increased AT2R expression and attenuation of AngII, AT1R, pERK1/2–NHE1–NCX signaling, oxidative stress, and inflammatory markers (ROS and NLRP3) in rats with MS. **Conclusion**: Our findings suggest that EMPA treatment is associated with improvement in selected local cardiac RAS components and modulation of the pERK1/2–NHE1–NCX signaling pathway, along with reduced oxidative stress, decreased inflammation, and improved cardiac function in MS.

## 1. Introduction

Cardiometabolic syndrome (CMS) is a multifaceted condition characterized by metabolic dysregulation, cardiovascular disease (CVD), and diabetes risk factors, with obesity as the primary trigger [1]. In the context of obesity and high-fat diet (HFD)-induced dyslipidemia, the local Renin–Angiotensin System (RAS) in the cardiovascular system becomes excessively activated [2]. The local RAS plays a significant role in cardiovascular health [3]. Its components are produced within the myocardium and can exert autocrine and paracrine effects. In the heart, the local RAS regulates myocardial contractility, cell growth, and extracellular matrix composition, all of which are essential for maintaining normal cardiac function [4]. Angiotensin 2 (AngII), a target molecule in the RAS, exerts both paracrine and autocrine roles within the heart [5]. Through AT1R (Angiotensin II type 1 receptor), AngII activates the downstream pERK1/2–NHE1–NCX signaling pathway [6], whereas engagement of AT2R (Angiotensin II type 2 receptor) leads to inhibition of pERK signaling [7]. In cardiac cells, increased NHE1 activity stimulates the reverse mode of NCX, leading to heightened NLRP3-mediated inflammation and increased oxidative stress due to elevated ROS levels [8].

Current therapies targeting the systemic RAS do not sufficiently modulate local RAS activity, highlighting the need for novel therapeutic strategies that specifically address the distinct roles of local RAS in cardiovascular disease [4,9]. Empagliflozin (EMPA), a sodium-glucose cotransporter 2 (SGLT2) inhibitor, lowers blood glucose by inhibiting reabsorption in the proximal tubules and increasing urinary glucose excretion [10]. Beyond its glycemic effects, EMPA modulates the local RAS by decreasing AT1R expression and increasing AT2R expression in the left ventricle, thereby mitigating oxidative stress [11]. Clinical evidence suggests that modulation of angiotensin pathways may contribute to the cardioprotective effects of EMPA [12]. Additionally, EMPA has been shown to reduce oxidative stress induced by AngII and hyperglycemic conditions in human cardiac tissues, which further supports its pleiotropic cardioprotective actions [13]. Furthermore, EMPA may attenuate oxidative stress and inflammatory responses by reducing ROS production and suppressing NLRP3 inflammasome activation, thereby contributing to its cardioprotective effects [14,15]. EMPA has also been shown to reduce AngII-induced cardiac hypertrophy by inhibiting the expression of SGLT1 and sodium–hydrogen exchanger 1 (NHE1) [16], and to suppress harmful local RAS components in the kidney [17]. However, some evidence suggests that the cardioprotective effects of EMPA are not mediated through direct inhibition of NHE1 [18].

A clinical trial demonstrated that EMPA significantly reduced the risk of major adverse cardiovascular events in patients with type 2 diabetes and established cardiovascular disease, including a 38% relative risk reduction in cardiovascular death and a 35% reduction in hospitalization for heart failure [19]. Despite these cardioprotective outcomes, evidence regarding EMPA’s effects on local RAS modulation and its relationship with NHE1 remains limited and somewhat contradictory. Some studies suggest that direct NHE1 inhibition may counteract EMPA’s protective actions, whereas others dispute this notion. Given that EMPA consistently decreases NHE1 expression, attention has shifted toward its upstream regulators, particularly components of the local RAS. Therefore, this study aimed to investigate the effects of EMPA on selected local cardiac RAS components and their association with pERK1/2–NHE1–NCX signaling, oxidative stress, and inflammatory responses in an experimental model of cardiometabolic syndrome.

## 2. Materials and Methods

### 2.1. Animals and Housing

Twenty-four healthy male albino Wistar rats (200–220 g) were obtained from the Department of Physiology, School of Medicine, Tehran University of Medical Sciences. Animals were housed in standard polycarbonate cages under controlled environmental conditions with the temperature maintained at 25 ± 2 °C, relative humidity between 45 and 55%, and a 12 h light/dark cycle (lights on at 07:00). All rats were fed a standard laboratory chow diet during the 7-day acclimatization period prior to experimental procedures and had ad libitum access to water. All experimental protocols were approved by the Ethics Committee of Tehran University of Medical Sciences (IR.TUMS.AEC.1402.018). All procedures involving animals were conducted in accordance with institutional guidelines of the NIH and complied with national regulations for the care and use of laboratory animals.

### 2.2. Experimental Design

Following a 7-day acclimatization period, rats were randomly assigned to two dietary groups:

1. Control group (*n* = 12): Standard rodent diet (10 kcal from fat).
2. High-fat diet group (*n* = 12): Specially formulated diet containing 60% kcal from fat that was purchased from Royan Institute for Biotechnology (Isfahan, Iran).

After 8 weeks, animals were further randomly divided into 4 groups (*n* = 6):

1. Control-Control (C-C): regular diet + vehicle treatment.
2. Control-EMPA (C-E): regular diet + EMPA (10 mg/kg/day).
3. Metabolic Syndrome-Control (MS-C): HFD + vehicle treatment.
4. Metabolic Syndrome-EMPA (MS-E): HFD + EMPA (10 mg/kg/day) [20].

A formal sample size calculation was not conducted. The sample size was determined based on prior comparable experimental studies and ethical considerations to reduce animal use. EMPA (Abidi Pharmaceutical Company, Tehran, Iran) was freshly suspended in sterile distilled water and gavaged daily (10 mg/kg). Throughout the 16-week study period (8 weeks HFD + 8 weeks intervention), all animals had ad libitum access to their respective diets and water, and body weight was measured weekly.

At the end of the experimental period, all animals underwent comprehensive terminal procedures. Cardiac function was first assessed by echocardiography under light anesthesia (sodium thiopental, 50 mg/kg, intraperitoneally). After a 48 h recovery period, the animals underwent a 12 h fast prior to final sample collection.

For euthanasia, deep anesthesia was induced with sodium thiopental (70 mg/kg, intraperitoneally). Blood samples were collected via abdominal aorta, allowed to clot for 30 min, and centrifuged at 3000× *g* for 15 min at 4 °C to separate serum, which was stored at −80 °C until biochemical analyses. Immediately after blood collection, the left ventricle was rapidly excised, briefly rinsed in ice-cold saline to remove residual blood, and snap-frozen in liquid nitrogen within 60 s. Tissues were stored at −80 °C for subsequent molecular analysis.

### 2.3. Echocardiography Assessment

Cardiac function was evaluated under light anesthesia (sodium thiopental 50 mg/kg, intraperitoneally) using a GE vivid 7 (GE Healthcare, Chicago, IL, USA) with a 12 MHz transducer. Parameters were measured by using 2D and M-mode echocardiography. Left ventricular posterior wall thickness in diastole and systole (LVPWD, LVPWS), interventricular septal thickness in diastole and systole (IVSD, IVSS), and left ventricular internal diameters in diastole and systole (LVIDd,s) were measured by placing the M-mode cursor perpendicular to the ventricular walls on the parasternal long-axis view. The endocardial border of the LV was traced in diastole and systole on B-mode transthoracic echocardiography images to calculate end-diastolic volume (EDV), end-systolic volume (ESV), and ejection fraction (EF). Stroke volume (SV) was calculated as EDV − ESV, and fractional shortening (FS) was calculated as FS = [(LVIDd − LVIDs)/LVIDd] × 100. Three consecutive cardiac cycles were analyzed by a blinded investigator, and animals maintained stable heart rates (300–400 bpm) during imaging. After echocardiography, rats were recovered with thermal support and subcutaneous saline.

### 2.4. Biochemical Analyses

Fasting blood sugar (FBS), total cholesterol, and triglycerides (TG) were measured using standard enzymatic colorimetric methods. Low-Density Lipoprotein-Cholesterol (LDL-C) and High-Density Lipoprotein-Cholesterol (HDL-C) levels were determined via direct enzymatic homogeneous assays (BioreX Fars kits, Shiraz, Iran). All analyses were performed using an Alpha Classic autoanalyzer (Audit Diagnostics, Cork, Ireland).

### 2.5. ELISA Protocol for ROS Measurement

All reagents, standards, and samples were prepared according to the manufacturer’s instructions for the Sunlong Biotech ROS ELISA Kit (Cat.No: SL- SL1189Ra, Hangzhou, China). After 20 min of equilibration at room temperature, standard (50 µL/well) or prepared samples (10 µL serum + 40 µL sample dilution buffer) were added to the antibody-precoated plate. The plate was incubated at 37 °C for 30 min, followed by 5 washes (300 µL/well) with the wash buffer. Next, 50 µL of HRP-conjugate was added to each well and incubated at 37 °C for 30 min. After another wash cycle (5 × 300 µL), 50 µL of Chromogen Solutions A and B (TMB substrate) were added and incubated in the dark at 37 °C for 15 min. The reaction was stopped with 50 µL stop solution, and absorbance was measured at 450 nm (reference 630 nm) using a microplate reader (BioTek Instruments, Winooski, VT, USA).

### 2.6. Western Blot Analysis

Western blot analysis was performed as previously described with minor modifications [21,22]. Tissues were lysed in radioimmunoprecipitation assay (RIPA) buffer. The lysates were removed by centrifugation at 14,000 rpm for 20 min at 4 °C. Protein concentration was determined using the Bradford Protein Quantification kit (DB0017, DNAbioTech, Tehran, Iran) according to manufacturer’s instructions. The tissue lysates were mixed with an equal volume of 2X Laemmli sample buffer. Lysates (20 μg) were then subjected to SDS-PAGE after a 5 min boiling and subsequently transferred to a 0.2 μm immune-Blot™ polyvinylidene difluoride (PVDF) membrane (Cat No: 162-017777; Bio-Rad Laboratories, Hercules, CA, USA). Next, the membranes were blocked with 5% BSA (Cat No: A-7888; Sigma-Aldrich, St. Louis, MO, USA) in 0.1% Tween 20 for 1 h. Then, the membranes were incubated with anti-AngII (Cat No: ABIN7445423, Antibodies, Aachen, Germany), anti- AT1R (Cat No: ab239995, Abcam, Cambridge, UK), anti-AT2R (Cat No: ab92445, Abcam, Cambridge, UK), pErk1/2 (Cat No: ab76299, Abcam, Cambridge, UK), NLRP3 (Cat No: ab263899, Abcam, Cambridge, UK), NHE1 (Cat No: ab67314, Abcam, Cambridge, UK), NCX1 (Cat No: ab177952, Abcam, Cambridge, UK), and anti-β actin-loading control antibodies (1/2500, Cat No: ab8227, Abcam, Cambridge, UK) for 1 h at room temperature. Subsequently, membranes were washed three times with TBST and incubated with goat anti-rabbit IgG H&L (HRP) (1/10,000, Cat No: ab6721; Abca, Cambridge, UK) secondary antibody. The membranes were then incubated with enhanced chemiluminescence (ECL) for 1–2 min. Protein expression was normalized to β-actin. Densitometry of protein bands was performed using the gel analyzer Version 2010a software (National Institutes of Health, Bethesda, MD, USA), such that the percentage area under the curve of each band was divided by the percentage area under the curve of its corresponding actin band. Calculated values were compared among groups as we described previously [23]. Western blot analyses were performed by laboratory personnel who were unaware of the treatment allocation.

### 2.7. Statistical Analysis

Statistical analyses were performed using GraphPad Prism (version 9). Data are reported as mean ± SEM. Statistical comparisons for quantitative data were performed using the one-way analysis of variance (one-way ANOVA) method. If significant differences were observed between groups, Tukey’s post hoc test was applied. Statistical significance was considered as *p* < 0.05.

## 3. Results

### 3.1. Effects of EMPA on Weight Gain, FBS and Lipid Profiles in MS

Induction of metabolic syndrome in the MS-C group led to significant metabolic alterations compared to the C-C group, including marked weight gain (57.40 ± 8.20 vs. 8.70 ± 8.90, *p* < 0.0001), elevated FBS (248 ± 19.36 vs. 106.20 ± 10.78, *p* < 0.0001) and dyslipidemia characterized by increased levels of TG (85.20 ± 5.9 vs. 28.82 ± 3.27, *p* < 0.0001) and LDL-C (30.58 ± 2.17 vs. 11.67 ± 1.15, *p* < 0.0001), accompanied by significantly reduced HDL-C levels (29.33 ± 1.91 vs. 44.50 ± 1.91, *p* < 0.0001). No significant increase in total cholesterol levels was observed in the MS-C group, indicating that the HFD did not substantially alter overall cholesterol homeostasis.

Treatment with EMPA significantly reduced weight gain (−17.20 ± 11.70 vs. 57.40 ± 8.20, *p* < 0.0001), decreased FBS (121.30 ± 7.22 vs. 248.19 ± 19.36, *p* < 0.0001) and improved lipid profiles, with reductions in TG (50.26 ± 5.63 vs. 85.20 ± 5.90, *p* = 0.0011) and LDL-C (21.80 ± 1.61 vs. 30.58 ± 2.17, *p* = 0.0042) levels as well as an increase in HDL-C (39.42 ± 1.72 vs. 29.33 ± 1.91, *p* = 0.002). The CE group showed no significant lipid profile changes, except for a slight LDL-C reduction (*p* = 0.016). EMPA treatment did not significantly affect total cholesterol levels in either group (Table 1).

### 3.2. Effects of EMPA on Cardiac Functional Parameters in MS

**Figure 1:**
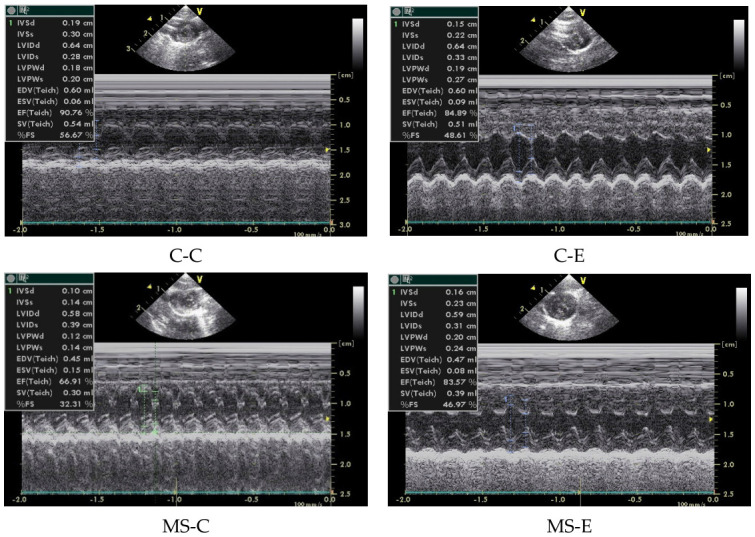
Representative echocardiographic images showing M-mode from the short axis view with example of calculation of the LV dimensions at baseline.

Echocardiographic results are summarized in Table 2. Although statistical analyses showed no significant differences among the groups in structural cardiac parameters—including IVSd, IVSs, LVIDd, LVIDs, LVPWd, LVPWs—functional cardiac indices were markedly impaired in the MS-C group compared with the C-C group. Specifically, the MS-C group exhibited significant reductions in SV (0.31 ± 0.02 vs. 0.52 ± 0.05, *p* = 0.0072), EF (69.16 ± 1.00 vs. 84.79 ± 1.4, *p* < 0.0001), and FS (34.36 ± 0.71 vs. 48.66 ± 1.60, *p* < 0.0001). Additionally, treatment with EMPA in the MS-E group resulted in a significant improvement in these functional parameters relative to the MS-C group (0.53 ± 0.04 vs. 0.31 ± 0.02, *p* = 0.0058, 77.67 ± 1.52 vs. 69.16 ± 1.00, *p* = 0.0002, 41.02 ± 0.41 vs. 34.36 ± 0.71, *p* = 0.0027) (Figure 1, Table 2).

### 3.3. Effects of EMPA on the Cardiac RAS in MS

**Figure 2:**
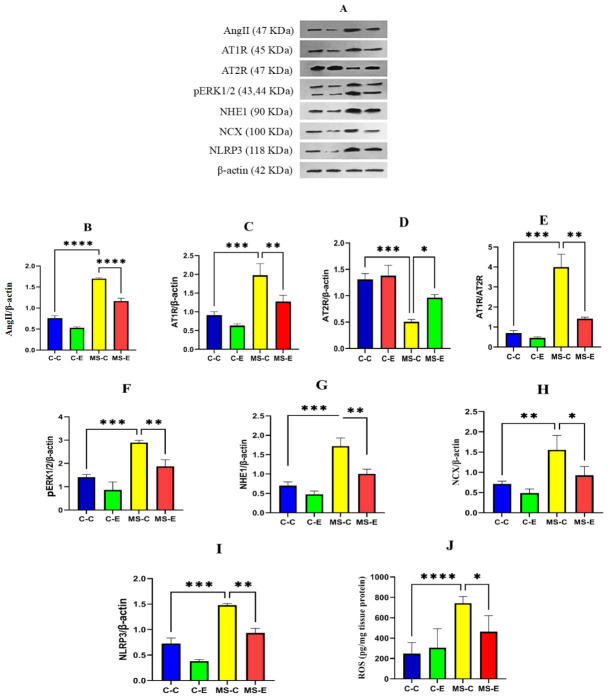
Effects of EMPA on the cardiac RAS and downstream signaling pathway in MS. (**A**) Representative Western blot bands for AngII, AT1R, AT2R, pERK1/2, NHE1, NCX, and NLRP3 are presented. Densitometric quantification was conducted using three independent biological samples per group (*n* = 3). Cardiac protein levels of (**B**) Angiotensin-2 (AngII)/β-actin, (**C**) Angiotensin type 1 receptor (AT1R)/β-actin, (**D**) Angiotensin type 2 receptor (AT2R)/β-actin, (**E**) AT1R-to-AT2R ratio, (**F**) Phosphorylated Extracellular Signal-Regulated Kinase 1/2 (pERK1/2)/β-actin, (**G**) Na+/H+ Exchanger 1 (NHE1)/β-actin, (**H**) Na+/Ca^2^+ Exchanger (NCX)/β-actin, (**I**) NOD-like receptor protein 3 (NLRP3)/β-actin inflammasome, (**J**) reactive oxygen species (ROS). Values are expressed as mean ± SEM (*n* = 3). Differences between groups were considered significant at *p* < 0.05 and were analyzed with one-way ANOVA followed by Tukey’s post hoc test. C-C: control, C-E: Control + EMPA, MS-C: Metabolic syndrome, MS-E: Metabolic syndrome + EMPA. * *p* < 0.05, ** *p* < 0.01, *** *p* < 0.001, **** *p* < 0.0001.

As shown in Figure 2, the MS-C group exhibited significantly increased cardiac AngII and AT1R levels compared with the C-C group (1.70 ± 0.02 vs. 0.75 ± 0.03, *p* < 0.0001 and 1.97 ± 0.17 vs. 0.91 ± 0.05, *p* = 0.0005, respectively) (Figure 2B,C). Conversely, AT2R protein levels were significantly decreased in the MS-C group compared to those in the C-C group (0.51 ± 0.04 vs. 1.31 ± 0.1, *p* = 0.0008) (Figure 2D), resulting in a markedly elevated AT1R/AT2R ratio (4 ± 0.63 vs. 0.7 ± 0.07, *p* = 0.0004) (Figure 2E). EMPA administration in the MS-E group reduced AngII and AT1R levels (1.16 ± 0.06 vs. 1.69 ± 0.02 *p* < 0.0001 and 1.27 ± 0.09 vs. 1.97 ± 0.17, *p* = 0.0068) (Figure 2B,C) and upregulated the AT2R levels (0.96 ± 0.05 vs. 0.51 ± 0.04 *p* = 0.0235) (Figure 2D), resulting in a lower AT1R/AT2R ratio (1.42 ± 0.07 vs. 4.00 ± 0.63, *p* = 0.0022) (Figure 2E).

### 3.4. Effects of EMPA on the Cardiac Expression of pERK1/2, NHE1 and NCX in MS

In the MS-C group, the expression levels of pERK1/2 were significantly elevated compared to the C-C group (2.89 ± 0.05 vs. 1.4 ± 0.06, *p* = 0.0003). Treatment with EMPA resulted in a significant reduction in the expression of pERK1/2 (1.87 ± 0.16 vs. 2.89 ± 0.05, *p* = 0.0033) (Figure 2F). Moreover, we observed a significant increase in NHE1 expression in the MS-C group relative to the C-C group (1.71 ± 0.12 vs. 0.70 ± 0.09, *p* = 0.0003), while the MS-E group exhibited a marked decrease in the levels of this exchanger (1.00 ± 0.06 vs. 1.71 ± 0.12, *p* = 0.0032) (Figure 2G).

NCX expression was also significantly increased in MS-C rats compared to the C group (1.55 ± 0.2 vs. 0.71 ± 0.04, *p* = 0.0062). In contrast, treatment with EMPA resulted in a notable decrease in NCX expression (0.92 ± 0.12 vs. 1.55 ± 0.20, *p* = 0.0298) (Figure 2H).

### 3.5. Effects of EMPA on the ROS and NLRP3 in MS

The MS-C group exhibited significantly elevated ROS levels and NLRP3 inflammasome expression compared with the C-C group (742.50 ± 26.87 vs. 247.80 ± 44.75, *p* < 0.0001 and 1.47 ± 0.01 vs. 0.72 ± 0.10, *p* = 0.001). Notably, EMPA treatment in the MS-E group effectively reduced both markers (462.60 ± 64.51 vs. 742.50 ± 26.87, *p* = 0.0110 and 0.93 ± 0.05 vs. 1.47 ± 0.01, *p* = 0.0013) (Figure 2I,J).

## 4. Discussion

This study showed that EMPA improves lipid metabolism, modulates the local RAS, and enhances cardiac function in a model of MS. These findings highlight the potential of EMPA as not only an antidiabetic agent but also a cardioprotective and metabolic modulator in MS-related complications.

HFD-induced MS resulted in significant dyslipidemia, indicated by elevated LDL-C and TG levels, reduced HDL-C levels, as well as increases in both FBS and weight gain, which aligns with previous reports on HFD models [15]. Notably, total cholesterol levels remained unchanged despite HFD intake, likely due to compensatory mechanisms, such as suppressed endogenous cholesterol synthesis, reduced intestinal absorption, and enhanced cholesterol elimination. These findings suggest that the liver possesses a notable capacity to regulate systemic cholesterol levels independently of lipoprotein metabolism [16].

### 4.1. EMPA Improves Dyslipidemia and Reduces Weight Gain and FBS in MS

EMPA treatment significantly reduced body weight gain and FBS, consistent with previous studies reporting the favorable metabolic effects of SGLT2 inhibitors [20]. While SGLT2 inhibitors, including EMPA, induce osmotic diuresis and a transient reduction in extracellular fluid volume, evidence from long-term studies indicates that these effects are not sustained and are largely compensated by physiological homeostatic mechanisms, rather than resulting in persistent hypovolemia [24]. This interpretation is further supported in our study by the absence of a significant difference in body weight between C-C and C-E groups. Moreover, EMPA treatment lowered LDL-C and TG while increasing HDL-C levels. Findings from animal studies regarding EMPA’s impact on lipid profile remain inconclusive, with variable outcomes likely due to the complexity of lipid metabolism [25,26]. In the present study, EMPA significantly reduced LDL-C levels compared with the MS-C group, although they did not return to those of the C-C group. Notably, the C-E group also exhibited higher LDL-C levels relative to the C-C group, suggesting that EMPA may elevate plasma LDL-C independently of dietary regimen. Nonetheless, the LDL-C reduction observed in the MS-E group indicates that HFD feeding substantially increased LDL-C levels in MS-C rats, and EMPA treatment partially normalized this elevation, resulting in values comparable to those of the C-E group.

Previous studies have reported that EMPA does not necessarily lower total LDL-C but selectively reduces small, dense LDL-C particles (atherogenic) and shifts the profile toward larger, less atherogenic particles [16]. This shift may explain why an increase in total LDL-C does not necessarily translate to higher atherogenic risk. In addition, EMPA has been shown to enhance insulin sensitivity, leading to reduced circulating insulin levels, activation of lipolysis, decreased TG, and increased HDL-C [26]. EMPA treatment did not significantly alter body weight in the C-E group, while LDL-C levels were significantly increased in the C-E group compared with the C-C group. These findings indicate that specific metabolic effects of EMPA may occur independently of changes in body weight. Consequently, the beneficial effects observed in the MS-E group are unlikely to be solely attributable to weight reduction.

### 4.2. Improvement in Cardiac Function with EMPA

Induction of MS impaired cardiac function, as reflected by reduced SV, EF, and FS, without significantly altering structural parameters (IVSd, IVSs, LVIDd, LVIDs, LVPWd, LVPWs). A longer duration of HFD exposure may be required to induce structural cardiac changes. These findings are consistent with prior reports demonstrating that HFD induces cardiac dysfunction [27,28]. EMPA treatment significantly improved SV, EF, and FS, in agreement with clinical evidence showing that SGLT2 inhibitors improve heart failure outcomes [29,30]. In our study, changes in EF were significant despite no change in LVIDd or LVIDs. This phenomenon may occur because variations in LVIDd and LVIDs have an usually wide range, but when included in a formula, they represent systolic function, as was observed in the study by Wang et al., where changes in EF were observed without changes in LVIDd and LVIDs [31].

### 4.3. Modulation of the RAS and Downstream Signaling

**Figure 3:**
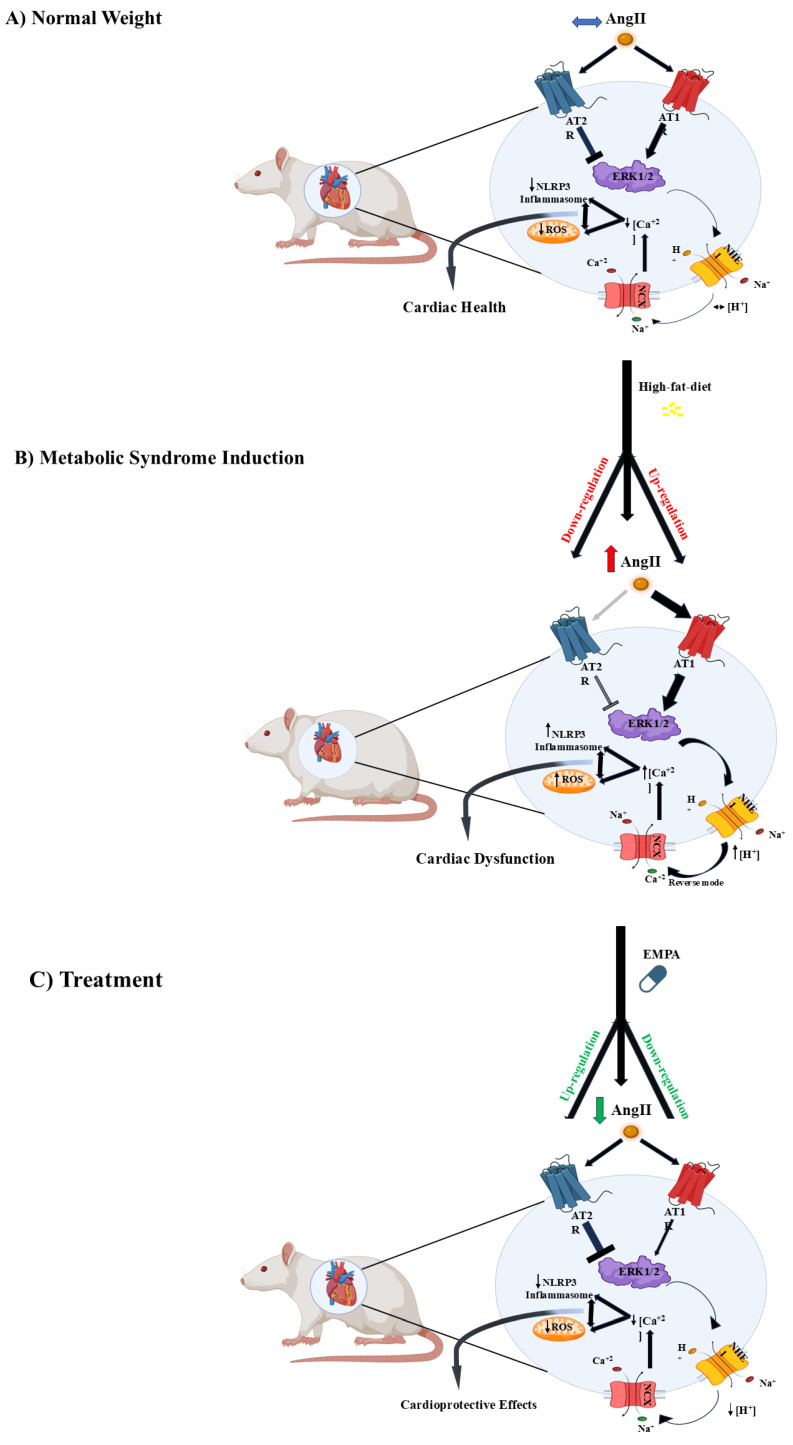
(**A**) When body weight is normal, circulating AngII level is low and AngII binds to AT2R, which mediates protective cardiovascular effects by inhibiting pERK1/2. (**B**) In the obese state characterized by chronic low-grade inflammation, this regulatory balance is disrupted and the subsequent binding of AngII to AT1R activates the pERK1/2-NHE1-reverse-mode NCX signaling pathway, leading to an elevation in intracellular Ca^2+^ concentration. Ca^2+^ overload drives excessive ROS production and activates the NLRP3 inflammasome, creating a pro-inflammatory cellular environment. (**C**) EMPA may exert cardioprotective effects by modulating RAS (increasing AT2R and decreasing AT1R) and downregulating downstream signaling molecules.

The MS-C group exhibited activation of the deleterious RAS pathway in cardiac tissue, characterized by significant upregulation of AngII and AT1R expression, while downregulating the protective AT2R receptor protein. This pattern is consistent with previous studies showing that oxidized LDL-C promotes pathological activation of the local cardiac RAS through AT1R overexpression [32]. Our findings also align with Moin’s work, which reported that in healthy conditions where AngII levels are low, ACE2 inhibits AT1R catalytic activity while facilitating AT2R-mediated protective effects. In chronic stress conditions such as MS, dissociation of AT1R from ACE2 leads to uncontrolled AT1R activation and its detrimental effects [33] (Figure 3A,B).

EMPA treatment reduced AngII and AT1R expression and increased AT2R expression, shifting the RAS axis toward greater cardioprotection. This shift likely improves cardiac function. The reciprocal changes in AT1R and AT2R are important, as their balance determines cardiac RAS activity. Restoring this balance may reduce downstream signaling and further enhance cardiac function following EMPA treatment.

### 4.4. EMPA Attenuates Oxidative Stress and Inflammation in Association with Modulation of the pERK1/2-NHE1-NCX Signaling Pathway in MS

MS rats showed significant upregulation of pERK1/2, NHE1, and NCX expression, along with increased ROS production and NLRP3 inflammasome activation. Various studies have shown that AngII, acting through AT1R, is known to activate pERK1/2, which subsequently upregulates NHE1 expression [21]. Downstream, NHE1 activation drives NCX reverse-mode activity, leading to elevated intracellular calcium. Increased cytosolic calcium promotes oxidative stress, stimulates ROS generation, and activates the NLRP3 inflammasome. These oxidative and inflammatory processes reinforce one another, exacerbating cardiac dysfunction [22]. Based on our findings, we suggest that in MS, dyslipidemia may contribute to a chronic increase in AngII levels, which may be associated with activation of the pERK1/2/NHE1/NCX pathway via AT1R, thereby promoting inflammation, oxidative stress, and subsequent cardiac dysfunction.

Conflicting findings have been reported regarding the effects of EMPA on NHE1 activity and expression. While one study demonstrated that EMPA directly inhibits NHE1 in cardiac cells [8], reduces NCX activity and thereby lowers cytosolic calcium, an effect that protects cardiomyocytes [34], and suppresses NLRP3 inflammasome activation and ROS production through a calcium-dependent mechanism [8,35], other evidence suggests the opposite outcome [18]. The observed decrease in NHE1 expression following EMPA treatment has shifted attention toward upstream regulatory pathways, particularly the RAS, given that NHE1 silencing itself protects against Ang II-induced cardiomyopathy [36]. The findings of the present study suggest that EMPA may be associated with reduced NHE1 expression by modulating the local cardiac RAS, ultimately contributing to cardioprotection by attenuating oxidative stress and inflammation and improving systolic function in metabolic syndrome.

## 5. Study Limitations

The present model represents a relatively short-term HFD-induced MS model. The HFD-fed rats developed obesity, hyperglycemia, and dyslipidemia, which are key metabolic features of obesity-associated MS. Blood pressure was not assessed in the present study; therefore, the presence or absence of hypertension, another important component of MS, could not be determined. The relatively short treatment duration limits the ability to evaluate the long-term effects of EMPA on cardiometabolic outcomes. Although EMPA improved the lipid profile, it did not fully normalize LDL-C levels, and its influence on body composition remains unclear. ROS levels were assessed using a commercial ELISA kit, which provides an indirect estimate of oxidative stress rather than a direct real-time measurement of intracellular ROS. Therefore, the ROS findings should be interpreted as an indirect indicator of oxidative stress rather than a direct quantification of intracellular ROS. Future studies incorporating complementary techniques, such as electron paramagnetic resonance (EPR/ESR) spectroscopy or ROS-sensitive fluorescent probes, may provide a more direct assessment of intracellular ROS and further validate the present findings. Additionally, intracellular Ca^2+^ levels, cardiac tissue pH, and several key components of the cardiac RAS—including ACE and ACE2 activity, Ang-(1–7) levels, and Mas receptor signaling—were not assessed in this study, which could have offered deeper insight into the drug’s cardioprotective mechanisms. Therefore, future studies should include measurement of these factors to provide a more comprehensive understanding of the cardiac RAS pathway and its underlying mechanisms. Another limitation of the present study is that the experimental design did not allow discrimination between the direct cardiac effects of EMPA and indirect effects secondary to improvements in metabolic parameters. Therefore, whether the observed molecular alterations represent primary mechanisms of EMPA action remains unclear. This study also focused solely on cardiac outcomes and included only male rats because female rodents exhibit ovarian hormonal fluctuations and relative resistance to HFD-induced metabolic disturbances, potentially increasing experimental variability. Consequently, sex-specific differences in the response to EMPA were not evaluated. Future studies including both sexes are warranted to clarify possible sex-dependent effects of EMPA treatment. Finally, the absence of a comparison between EMPA and an NHE1 inhibitor, either alone or in combination, precludes conclusions about potential synergistic or independent effects.

## 6. Conclusions

Our findings suggest that EMPA treatment is associated with partial restoration of the balance between deleterious and protective components of the local cardiac RAS, modulation of the pERK1/2–NHE1–NCX signaling pathway, reduced oxidative stress and inflammation, and improved cardiac function in metabolic syndrome. These findings indicate that EMPA may represent a promising adjunctive therapeutic option for attenuating cardiometabolic complications associated with MS.

## Figures and Tables

**Table 1:** Effects of EMPA on weight gain, FBS, and lipid profile (total cholesterol, LDL-C, HDL-C and TG) in MS.

	Groups	C-C	C-E	MS-C	MS-E
Parameters					
Weight Gain, Week 8–16 (gr)		8.7 ± 8.9	8.8 ± 9.8	57.4 ± 8.2 **	−17.2 ± 11.7 ^####^
FBS (mg/dL)		106.2 ± 10.78	115 ± 12.14	248.7 ± 19.36 ****	121.3 ± 7.23 ^####^
Total Cholesterol (mg/dL)		50.42 ± 2.56	58.00 ± 2.9	63.83 ± 4.04	57.25 ± 4.16
LDL-C (mg/dL)		11.67 ± 1.16	19.42 ± 0.96 *	30.58 ± 2.17 ****	21.8 ± 1.61 ^##^
HDL-C (mg/dL)		44.50 ± 1.91	38.22 ± 1.79	29.33 ± 1.31 ****	39.42 ± 1.72 ^##^
TG (mg/dL)		28.82 ± 3.27	42.83 ± 5.53	85.2 ± 5.90 ****	50.26 ± 5.63 ^##^

Values are presented as mean ± SEM (*n* = 6). Differences between groups were considered significant at *p* < 0.05 and were analyzed with one-way ANOVA followed by Tukey’s post hoc test. C-C: control, C-E: Control + EMPA, MS-C: Metabolic Syndrome, MS-E: Metabolic Syndrome + EMPA, FBS: Fasting Blood Sugar, LDL-C: Low Density Lipoprotein-Cholesterol, HDL-C: High Density Lipoprotein-Cholesterol, TG: Triglycerides. * *p* < 0.05, ** *p* < 0.01 and **** *p* < 0.0001 compared with the C-C group, ^##^ *p* < 0.01 and ^####^ *p* < 0.0001 compared with the MS-C group.

**Table 2:** Echocardiographic findings of HFD-induced MS rats treated with EMPA.

	Groups	C-C	C-E	MS-C	MS-E
Parameters					
IVSd (cm)		0.19 ± 0.02	0.17 ± 0.00	0.14 ± 0.01	0.17 ± 0.00
IVSs (cm)		0.24 ± 0.02	0.24 ± 0.01	0.18 ± 0.01	0.20 ± 0.01
LVIDd (cm)		0. 64 ± 0.02	0.58 ± 0.01	0.58 ± 0.01	0.64 ± 0.02
LVIDs (cm)		0.33 ± 0.02	0.32 ± 0.00	0.38 ± 0.01	0.37 ± 0.02
LVPWd (cm)		0.18 ± 0.01	0.20 ± 0.01	0.18 ± 0.02	0.18 ± 0.01
LVPWs (cm)		0.22 ± 0.01	0.23 ± 0.02	0.22 ± 0.02	0.23 ± 0.01
EDV (mL)		0.62 ± 0.06	0.50 ± 0.04	0.53 ± 0.08	0.68 ± 0.06
ESV (mL)		0.1 ± 0.01	0.08 ± 0.00	0.15 ± 0.01 *	0.15 ± 0.02
SV (mL)		0.52 ± 0.05	0.42 ± 0.03	0.31 ± 0.02 **	0.53 ± 0.04 ^##^
EF (%)		84.79 ± 1.4	82.25 ± 1.1	69.16 ± 1.0 ****	77.67 ± 0.52 ^###^
FS (%)		48.66 ± 1.6	47.94 ± 1.7	34.36 ± 0.71 ****	41.02 ± 0.41 ^##^

Values are presented as mean ± SEM (*n* = 6). Differences between groups were considered significant at *p* < 0.05 and were analyzed with one-way ANOVA followed by Tukey’s post hoc test. C-C: control, C-E: Control + EMPA, MS-C: Metabolic Syndrome, MS-E: Metabolic Syndrome + EMPA. IVSd, Interventricular Septal thickness in Diastole; IVSs, Interventricular Septal thickness in Systole; LVIDd, Left Interventricular Internal Dimension in Diastole; LVIDs, Left Ventricular Internal Dimension in Systole; LVPWd, Left Ventricular Posterior Wall in Diastole; LVPWs, Left Ventricular Posterior Wall in Systole; EDV, End Diastolic Volume; ESV, End Systolic Volume; EF, Ejection Fraction; FS, Fraction Shortening. * *p* < 0.05 and ** *p* < 0.01 and **** *p* < 0.0001 compared with the C-C group, ^##^ *p* < 0.01 and ^###^ *p* < 0.001 compared with the MS-C group.

## Data Availability

The datasets generated during the current study are available from the corresponding authors on reasonable request.
